# Composite Hydrogels of Ultrasound-Assisted-Digested Formic Acid-Decellularized Extracellular Matrix and Sacchachitin Nanofibers Incorporated with Platelet-Rich Plasma for Diabetic Wound Treatment

**DOI:** 10.3390/jfb14080423

**Published:** 2023-08-11

**Authors:** Chien-Ju Lin, Hong-Liang Lin, Wen-Chen You, Hsiu-O Ho, Ming-Thau Sheu, Ling-Chun Chen, Wei-Jie Cheng

**Affiliations:** 1 School of Pharmacy, College of Pharmacy, Kaohsiung Medical University, Kaohsiung 80708, Taiwan; mistylin@kmu.edu.tw (C.-J.L.); hlglin@kmu.edu.tw (H.-L.L.); 2 School of Pharmacy, College of Pharmacy, Taipei Medical University, Taipei 11031, Taiwanhsiuoho@tmu.edu.tw (H.-O.H.); mingsheu@tmu.edu.tw (M.-T.S.); 3 Department of Biotechnology and Pharmaceutical Technology, Yuanpei University of Medical Technology, Hsinchu 30015, Taiwan

**Keywords:** ultrasound, decellularized extracellular matrix, platelet-rich plasma, sacchachitin, wound dressing, hydrogel

## Abstract

In this study, an ultrasound-assisted digestion method of a formic acid-decellularized extracellular matrix (dECM) of porcine skin was developed and optimized to form UdECM hydrogels for diabetic wound healing. Results demonstrated that ultrasonication improved the extraction rate of collagen from dECM samples, preserved the collagen content of dECM, reduced residual cells, and extracted greater DNA contents. Scanning electron microscope (SEM) analyses were performed, which demonstrated the optimal porosity on the surface and density of the cross-section in the hydrogel structure, which could control the release of growth factors embedded in UdECM hydrogels at desirable rates to boost wound healing. A wound-healing study was conducted with six different composite hydrogels, both empty materials and materials enriched with rat platelet-rich plasma (R-PRP), sacchachitin nanofibers (SCNFs), and TEMPO-oxidized sacchachitin in diabetic rats. The assessment based on scars stained with hematoxylin and eosin (H&E), Masson’s trichrome (MT), and a cluster of differentiation 31 (CD31) staining showed that the UdECM/SC/R-PRP treatment group had the most significant efficacy of promoting healing and even recovery of diabetic wounds to normal tissues. UdECM/R-PRP and UdECM/SCNFs demonstrated better healing rates than UdECM hydrogel scaffolds, which had only recovered 50% resemblance to normal skin. Treatment with both UdECM/TEMPO 050 and UdECM/TEMPO 050/R-PRP hydrogel scaffolds was ranked last, with even poorer efficacy than UdECM hydrogels. In summary, formulated UdECM and SCNF hydrogels loaded with PRP showed synergistic effects of accelerating wound healing and ultimately stimulating the wound to recover as functional tissues. This newly UdECM/SCNF composite hydrogel has promising potential for healing and regenerating diabetic wounds.

## 1. Introduction

Extracellular matrices (ECMs) obtained from decellularized tissues have emerged as a highly promising approach for tissue engineering scaffolds and acellular regenerative strategies [1,2,3,4,5]. Notably, decellularized (d)ECM can undergo enzymatic digestion to form inducible hydrogels, facilitating minimally invasive delivery through injection to target tissues [6,7,8,9]. These degradable dECM hydrogels are prosurvival, immunomodulatory, and vascularized, among a host of other pro-regenerative functions [2,10,11,12]. The most prevalent method to produce dECM hydrogels is via pepsin-mediated (stirred in pepsin with dilute hydrochloric acid) solubilization from the comminuted (powder) form of dECM (also called “ECM digestion”) as reported by Freytes et al. (designated herein as the “Freytes method”) [6]. Another method involves the use of 0.5 M acetic acid instead of 0.1 M HCl as the base medium for the pepsin enzyme (designated the “Voytik-Harbin method”) [13]. Alternatives include an extraction process to solubilize and form ECM hydrogels from soft tissues (designated the “Uriel method”) [14,15]. Recently, a new method was reported by Hussey et al. to prepare hydrogels from ECM bioscaffolds by rapid ultrasonic (US) cavitation without acidic or alkaline solutions, protease digestion, or chemical extraction and dialysis [16]. The US cavitation method described herein claimed to produce marked improvements in rheological properties and processing time over traditional enzymatic methods.

In order to obtain dECM for dECM hydrogels, a simple and effective decellularization technique was developed and optimized to attain dECM from porcine skin in our previous study [17]. dECM hydrogels were further fabricated by digesting the so-obtained dECM powder with pepsin in various acidic solutions and then treatment with pH-controlled neutralization and a temperature-controlled gelation procedure. The study on wound healing in diabetic patients and the histological examinations demonstrated that the combination of resultant dECM hydrogels and sacchachitin nanofibers (SCNFs) in composite hydrogels effectively expedited the healing process of diabetic wounds. Additionally, this synergistic approach stimulated the regeneration of hair follicles and sweat glands, resulting in the restoration of fully functional tissues. Although formic acid-decellularized pepsin-soluble ECM hydrogels were effective for treating diabetic wounds, the process still was time-consuming and less effective for retaining collagen and glycosaminoglycan (GAG). Overall, there is an unmet need to develop simple, effective, and optimized decellularization techniques and fabrication to obtain dECM hydrogels from porcine skin for wound-healing medical applications.

Studies have shown that US can increase collagen production by up to 124% and significantly reduce extraction times compared to traditional pepsin isolation methods. Further, the triple helix structure of collagen remains intact with US extraction. Finally, because US can promote the dispersion of large enzyme aggregates and widen the contact area with collagen fibers for hydrolysis, it further improves the digestion of enzyme molecules only on the surface of collagen fibrils [18]. In conclusion, gentle US for collagen extraction can optimize the extraction capacity without affecting the collagen content.

A previous study showed that SCNFs generated by NanoLyzer^®^ and TEMPO-oxidation possessed a gel-forming property to act as a biomaterial with ideal characteristics [19]. For diabetic wound-healing applications, both mechanically disintegrated SCNFs (SCN5) and TEMPO-oxidized sacchachitin nanofibers (T050SC; TEMPO 050) accelerated wound-healing and formed nearly identical normal tissues. As for T050SC/H, it further boosted the growth of sweat glands and hair follicles and had the ability to rebuild wounds into functional tissues. Furthermore, multifunctional biomaterials based on SCNFs might have great potential for future clinical applications. Some studies also showed that wound healing can be accelerated with platelet-rich plasma (PRP), which is enriched from blood and usually has 3–7 times the average platelet concentration of whole blood. After activation, greater amounts of growth factors, such as platelet-derived growth factor (PDGF), vascular endothelial growth factor (VEGF), transforming growth factor (TGF)-β, and epidermal growth factor (EGF), can be released. It not only stops bleeding but also inhibits cytokines and inflammatory symptoms, promotes the formation of microvessels, and accelerates the epithelialization of chronic wounds, thereby stimulating wound healing and tissue regeneration [20,21].

In this study, we hypothesized that the combination of the acidic addition and ultrasonication could improve the preparation of dECM hydrogel. This approach aims to reduce decellularization and fabrication time while increasing the extraction rate of collagen and related glycosaminoglycans (GAGs). Furthermore, we incorporated SCNFs or TEMPO 050 to form a composite hydrogel, which carried PRP and was expected to demonstrate a synergistic wound-healing effect on diabetic wounds.

## 2. Materials and Methods

### 2.1. Materials

The protein marker was purchased from BioTools (New Taipei, Taiwan). Tetramethyl ethylenediamine (TEMED), ammonium persulfate (APS),30% acrylamide/bis solution (37.5:1), and glycine were purchased from Bio-Rad (Hercules, CA, USA). Sodium chloride (NaCl) and sodium hydroxide (NaOH) were purchased from Showa Chemical (Tokyo, Japan). Trypsin, pepsin, formic acid, 10% formaldehyde, papain, Trizma^®^ base, L-cysteine hydrochloride, sodium EDTA, sodium dodecyl sulfate (SDS), acetic acid, glycerol, bromophenol blue, 2-mercaptoethanol, methanol, Coomassie blue R-250, and nicotinamide (Vitamin B3) were bought from Sigma-Aldrich (St. Louis, MO, USA). The glycosaminoglycan kit and hydroxyproline kit were obtained from Chondrex (Redmond, WA, USA). Penicillin/streptomycin (PS) and phosphate-buffered saline (PBS) were obtained from Corning (Corning, NY, USA). Triethanolamine (TEA) and hydrochloric acid (HCl) were purchased from Merck (Darmstadt, Germany). Porcine type I collagen standard-FlexiCol^®^ was bought from Advanced BioMatrix (San Diego, CA, USA). SC (2% *w*/*v* hydrogel), prepared by the mechanical disintegration of SCNFs in double-distilled (dd)H_2_O, was previously reported [19]. Fetal bovine serum (FBS), minimum essential medium (MEM), and a PicoGreen quantification kit were purchased from Thermo Fisher Scientific (Waltham, MA, USA). Streptozotocin (STZ) was bought from ChemCruz^®^ (Santa Cruz, CA, USA). An anti-cluster of differentiation 31 (CD31) antibody was obtained from Abcam (Cambridge, UK). Rat (R)-PRP was provided by the laboratory of Prof. Wu Yina, Department of Medicine, Fu Jen University (New Taipei City, Taiwan). Fresh white-fur pigskin was purchased from Wuxing Traditional Market (Taipei, Taiwan). Attane (isoflurane) was supplied by Panion & BF Biotech (New Taipei City, Taiwan). A mouse TGF-β1 DuoSet enzyme-linked immunosorbent assay (ELISA) (DY1679-05) and Mouse/Rat PDGF-AB DuoSet ELISA (DY8460-05) were purchased from R&D Systems (Minneapolis, MN, USA). A Quant-iT™ PicoGreen™ dsDNA assay kit (Molecular Probes, P7589) was provided by Thermo Fisher Scientific.

### 2.2. Methods

#### 2.2.1. Preparation of US-Assisted Pepsin-Solubilized dECM Hydrogels

Pretreatment of porcine skin to obtain dECM fragments followed the method developed by Hsieh et al. [17]. After ground dECM fragments were passed through a #40 screen, 250 mg of dECM plus 1 mg of pepsin (250 U/mg) were extracted in a 50 mL centrifuge tube with 10 mL of 0.5 M acetic acid. US was performed to assist pepsin solubilization in a Q Sonica (Q700) US breaker with a 1/8” probe immersed at the 4 cm position in the centrifuge tube. The operational parameters were set with a 2 s on-time followed by a 3 s off-time. The frequency and power settings were 24 kHz and 150 W, respectively. Over a duration of 5 min, the cumulative energy delivered amounted to 2300 J. After extraction, 1.0 M NaCl was added to salt out for 12 h, then it was centrifuged to remove the supernatant, and the precipitate was redissolved in 10 mL of a 0.5 M acetic acid solution. Afterward, the solution was loaded onto a dialysis membrane with a molecular weight (MW) cutoff of 3000 and subjected to dialysis against a 0.1 M acetic acid solution for a duration of 2 days. Finally, ddH_2_O was added for the final dialysis until neutral, and the solution was lyophilized to obtain UdECM. Then, 25, 50, and 75 mg of UdECM were weighed in 5 mL centrifuge tubes, and 0.5 M acetic acid was added. After being completely dissolved, the pH value was adjusted to neutral (pH 7.0) with 2 N NaOH to obtain UdECM hydrogels, which were respectively designated UdECM25, UdECM50, and UdECM75.

#### 2.2.2. Biochemical Characterization of UdECM Hydrogels

##### Qualitative Analysis of Collagen

In order to characterize the collagen in the UdECM hydrogels, SDS-polyacrylamide gel electrophoresis (PAGE) was conducted using 5% (*w*/*v*) polyacrylamide for the separating gel and 4% (*w*/*v*) polyacrylamide for the stacking gel. UdECM hydrogels and porcine type I collagen standard were prepared in 0.5 M Tris-HCl buffer (pH 6.8) containing 30% glycerol, 10% SDS, 1% 2-mercaptoethanol, and 0.02% bromophenol blue. The samples were heated to 95 °C for 5 min to ensure proper denaturation. Subsequently, 8 µL of a protein marker, 30 µL of the standard, and 30 µL of hydrogels were loaded into the gel. The electrophoresis was conducted at 85–100 V on vertical slab gels until the bromophenol blue front reached the bottom of the gel. The polyacrylamide gels were stained for 2 h with 0.1% Coomassie blue R-250 in acetic acid/methanol/water (2:5:5, *v*/*v*/*v*). The gels were then destained in a solution containing 7.5% acetic acid/15% methanol. Following the staining process, the stained polyacrylamide gels were recorded using an iPhone camera (Apple, Cupertino, CA, USA)

##### Quantitative Analysis of Collagen

Collagen contents in UdECM were determined with a Hydroxyproline assay kit (#6017, Chondrex, Woodinville, WA, USA). Samples were prepared according to the instructions with the assay kit as follows: UdECM hydrogels were hydrolyzed with 10 N hydrochloric acid and reacted with chloramine-T for 20 min. Next, a p-dimethylaminobenzaldehyde solution was added, and the resultant mixture was heated to 60 °C for 30 min. After reacting, the absorbance value was measured at a wavelength of 530 nm, and the amount of hydroxyproline was calculated by an automated imaging system and multifunctional optical detector (Cytation 3™, Cell Imaging Multi-Mode Reader, BioTek, Winooski, VT, USA). The total amount of collagen was calculated based on the following formula with the so-obtained hydroxyproline level:

(1) $$Hydroxyproline\;level\left(\frac{\mu g}{mg}\right)\times \frac{100}{13.5}=Collagen\;level\left(\frac{\mu g}{mg}\right)$$

##### DNA Quantitative Analysis

In order to quantify the amount of residue DNA in UdECM, the PicoGreen dsDNA detection kit was used. Sample preparation was performed according to the manufacturer’s instructions. Subsequently, the fluorescence intensity was measured using the Cytation 3 cell imaging multi-mode reader (BioTek) with an excitation wavelength of 480 nm and an emission wavelength of 520 nm. To subtract the background fluorescence, the fluorescence of the DNA-free blank was conducted and subtracted from the fluorescence of the experimental groups, ensuring accurate measurement of the sample’s fluorescence signal.

#### 2.2.3. Physical Characterizations of UdECM Hydrogels

##### Rheological Studies of UdECM Hydrogels

We evaluated the rheological characteristics of different concentrations (25, 50, and 75 mg/mL) of UdECM (UdECM25, UdECM50, and UdECM75) at 25 and 37 °C as previously reported [22]. We tested the oscillatory shear stress by the same procedure as previously reported [23], and the Amplitude Sweep program in the intelligent advanced rheometer (MCR 102e Anton-Paar, St. Albans Hertfordshire, UK) was used to analyze the UdECM25, UdECM50, and UdECM75 hydrogels, which yielded values for G′ (storage modulus) and G″ (loss modulus). The elastic modulus was measured by placing 0.3 mL of a hydrogel sample on rheometer plates at 25 and 37 °C with a fixed amplitude frequency of 1 Hz and a stress (strain) range of 0.1–100 Pa. Plotting G′ on the X-axis as and G″ on the Y-axis, the linear viscoelastic region (LVR) was obtained to numerically analyze the colloidal stability of the sample.

##### Morphological Observations

Freeze-dried UdECM25, UdECM50, UdECM75, and UdECM/SC sponges were mounted with conductive carbon tape, which was sputter-coated with gold (Hitachi IB-2, Tokyo, Japan). The images were assessed by Hitachi SU3500 scanning electron microscopy (SEM) at a 5 mm working distance and an accelerating voltage of 2.5 kV. The surface morphology and internal pores were observed for different concentrations of those UdECM hydrogels.

#### 2.2.4. R-PRP Preparation and Determination of PDGF-AB and TGF-β1

In order to improve the ability of wound-healing, R-PRP provided by the laboratory of Prof. Wu Yina of the Department of Medicine of Fu Jen University (New Taipei City, Taiwan) was incorporated with composite hydrogels as an adjuvant for treatment of diabetic wounds in animals. In this experiment, the commercially available enzyme immunosorbent assay kits (#DY1679-05, DY8460-05, R&D Systems) were used in an ELISA to determine the contents of PDGF-AB and TGF-β1 in R-PRP. The preparation method of the standard and test product was based on the attached instructions. Furthermore, the automated imaging system and multifunction optical detector (Cytation™ 3 Cell Imaging Multi-Mode Reader, BioTek) measured the absorbance at a wavelength of 450 nm, and PDGF-AB and TGF-β1 contents were calculated. Each test had two replicates, and an average value was obtained.

#### 2.2.5. In Vivo Studies of Diabetic Wound-Healing

##### Preparation of Wound Dressings for Animal Studies

Wound dressings were formulated in the proportions shown in Table 1. Formulation A (5% UdECM hydrogel, UdECM50) contained 50 mg of UdECM completely dissolved in 1.0 mL of 0.5 M acetic acid, and the pH was adjusted to neutral (pH 7.0) with 0.2 N NaOH. Formulation B (UdECM/PRP) had 800 pg of R-PRP added to 1.0 mL of formulation A and evenly mixed; it was then placed in a refrigerator at 4 °C for later use. Formulation C (UdECM/SCNF hydrogel) consisted of 50 mg of SCNFs dispersed in 1.0 mL of formulation A and mixed until it had formed a hydrogel. Formulation D (UdECM/SCNF/R-PRP) had 800 pg of R-PRP added to 1.0 mL of formulation C and evenly mixed; it was then placed in a refrigerator at 4 °C for later use. Formulation E (UdECM/TEMPO 050) had 50 mg Temp50 dispersed in 1.0 mL of formulation A and mixed until it had formed a hydrogel. Formulation F (UdECM/TEMPO 050/PRP) had 800 pg of R-PRP added to 1.0 mL of formulation E and evenly mixed; it was then placed in a refrigerator at 4 °C for later use.

##### Laboratory Animals

This animal experiment and experimental protocol were approved by the Institutional Animal Care and Use Committee of Taipei Medical University (Approval No.: LAC-2021-0024) in compliance with the Taiwanese Animal Welfare Act. Sprague-Dawley (SD) male rats at approximately 280–290 g and 8 weeks of age were purchased from BioLASCO (Taipei, Taiwan). Experimental rats were kept at 22–25 °C and a humidity of 60–70% with free intake of aseptic standard feed and drinking water under 12 h of automatic light and dark control. The experimental animals were given sufficient time to acclimate to the environment, allowing their physiological and psychological states to stabilize before conducting the experiment.

##### Evaluation of Induction of Type 1 Diabetes of the Animal Model

In this experiment, rats were raised for 1 week to achieve more than 300 g in weight, and then 180 mg/kg of a 50% nicotinamide solution (nicotinamide, NAM, vitamin B3) was given by an intraperitoneal (IP) injection. After 15 min, the animal model of type 1 diabetes was established by an IP injection of streptozotocin (STZ) (65 mg/kg) dissolved in 0.1 M citrate buffer (pH 4.5). Because STZ can cause large amounts of pancreatic insulin to be released and lead to fatal hypoglycemia, it is important to pay attention to any adverse effects and deaths over the next 24 h. The weight was measured, and blood glucose was detected by ACCU-CHEK^®^ (Performa #06870244, Roche, Basel, Switzerland) on days 3, 5, and 7 after induction. Rats exhibiting moderate hyperglycemia (i.e., blood glucose concentration of >250 mg/dL) and typical symptoms of diabetes (eating more, drinking more, frequent urination, and weight loss) were selected for the experiments [24].

##### Experimental Design and Surgical Methods

**Scheme 1:**
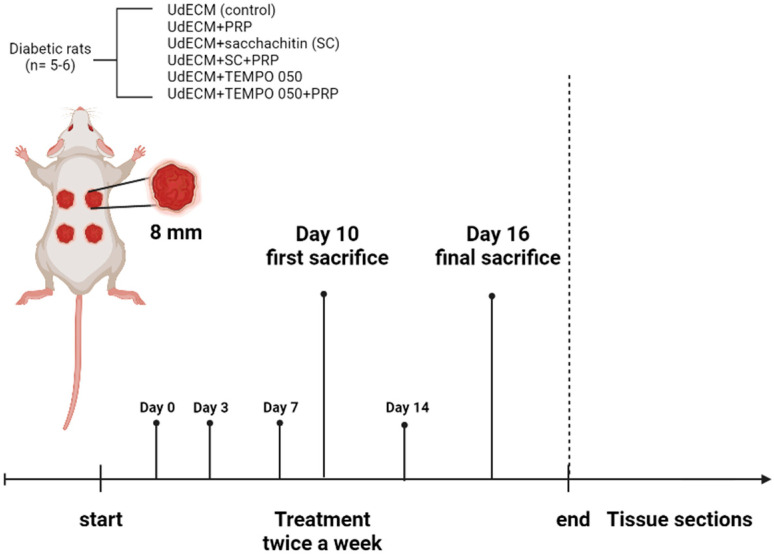
Experimental schedule of the animal experiment of diabetic rats.

Following the protocol shown in Scheme 1, rats with successful induction of diabetes were divided into six groups (*n* = 5 or 6) and IP injected with 10 mg/kg of Zoletil^®^ to anesthetize them. The back hair was removed, and an 8 mm tissue sampler was used to create a wound. Each rat had four holes drilled into its back, and a silicone sheet was glued with instant glue around the wound. Next, the silicone sheet was fixed onto the skin with nylon thread sutures. Then, 0.28 g of different wound dressings (UdECM, UdECM/PRP, UdECM/SCNFs, UdECM/SCNFs/PRP, UdECM/TEMPO 050, UdECM/TEMPO 050/PRP) were placed on the wound, followed by breathable film fixation, and finally being dressed in elastic bandages.

##### Analysis of Wound-Healing

After wound establishment, we changed the hydrogel dressings about every 3 days (on days 3, 7, 10, 14, 16, and 20). Furthermore, we took images of the wound, monitored blood glucose, and recorded the weight every day. ImageJ software (National Institutes of Health, Bethesda, MD, USA) was used to calculate the wound area and assess wound healing.

##### Histopathological Biopsy Evaluation

On days 10 and 16 post-treatment, rats were euthanized with excess CO_2_. Then, the skin of the wound was cut and fixed with 10% formaldehyde solution for 24 h. Paraffin-embedding and sectioning treatment were entrusted to Toson Technology (Zhubei City, Hsinchu County, Taiwan). Tissue sections were stained with hematoxylin and eosin (H&E) and Masson’s trichrome (MT) stain to assess collagen regeneration and, with CD31 endothelial cell markers (Abcam, ab182981), to evaluate the degree of angiogenesis. After sealing the film, skin tissue recovery was observed by light microscopy (BX43, Olympus, Center Valley, PA, USA).

#### 2.2.6. Statistical Analysis

Statistical analyses were performed using one-way analysis of variance (ANOVA) along with Bartlett’s statistical correction. A significance level of *p* < 0.05 was considered to indicate a statistically significant difference. The significance levels *p* < 0.05, *p* < 0.01, and *p* < 0.005 were denoted by *, **, and ***, respectively.

## 3. Results and Discussion

### 3.1. Preparation and Characterization of UdECM Hydrogels

#### 3.1.1. UdECM Process Development

**Figure 1:**
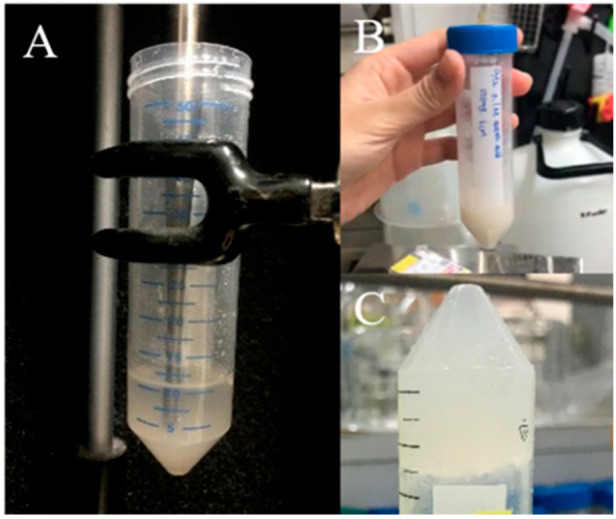
(**A**) The 1/8” probe tip was dipped into the 4 cm position of the 50 mL centrifuge tube for ultrasound (US) treatment. (**B**) US-assisted pepsin-solubilized decellularized extracellular matrix (UdECM) was ground and sifted through a #40 mesh screen, then US processed into UdECM hydrogels at low temperature. (**C**) Inversion of a UdECM hydrogel in a centrifuge tube to confirm hydrogel formation.

dECM was decellularized with 30% formic acid and made into physically crosslinked UdECM hydrogels with US. The collagen content was analyzed, and then rheology and morphology were used to examine the UdECM colloidal type. In a previous experiment, we found that dECM solubilized by citric acid at various concentrations was unable to form UdECM hydrogels above 25 °C. Therefore, we solubilized dECM with acetic acid. We sieved granular dECM through a #40 screen and dissolved it in 1 mg/mL pepsin (250 U/mg in a 0.5 M acetic acid solution); also, the US setup illustrated in Figure 1A was optimized to form UdECM hydrogels at 25 mg/mL dECM in a 0.5% acetic acid solution. After US and neutralization, as shown in Figure 1B,C, the UdECM solution could transform into UdECM hydrogels at room temperature. As reported previously, the collagen fiber could self-assemble into bundled fibers that physically crosslink to form the hydrogel at neutral pH [25]. Additionally, hydrolyzed collagen was also found to form hydrogels when cooled to temperatures below 30 °C. The phenomenon is similar to the thermomechanical properties of UdECM hydrogels treated by sonication [26]. Therefore, this result demonstrated that the application of ultrasonication could effectively extract collagen proteins and subsequently facilitate their self-assembly to form hydrogels.

#### 3.1.2. Qualitative and Qualitative Analysis of Collagen in UdECM

**Figure 2:**
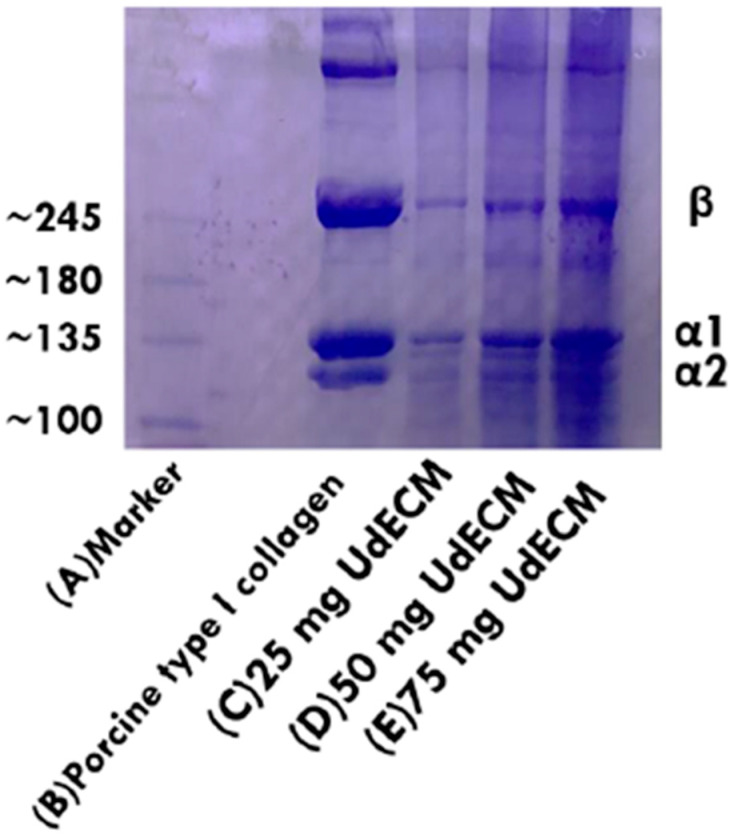
SDS-PAGE electrophoresis patterns. A, Marker; B, porcine type I collagen standard; C, ultrasound-assisted pepsin-solubilized decellularized extracellular matrix at 25 mg/mL (UdECM25); D, UdECM50; E, UdECM75.

SDS-PAGE analysis was used to evaluate collagen qualities for three groups of UdECM hydrogels (UdECM25: 25 mg/mL, UdECM50: 50 mg/mL, and UdECM75: 75 mg/mL), and results are shown in Figure 2. Typically, after heating the UdECM with SDS to unfold and linearize the protein structures by denaturation, type I collagen should show two different α-chains (two α1-chains and one α2-chain), two different β-chains (with crosslinks between two α1-chains or one α1-chain and one α2-chain), and a single γ-chain (with crosslinks between three α-chains). Different combinations of α-chains (two α1-chains and one α2-chain) comprised type I collagen fibers. As illustrated in Figure 2, the leftmost lane A of SDS-PAGE is a protein marker with molecular weights of 245, 180, 135, 100, 75, and 63 kDa from top to bottom, while lane B is a type I collagen standard, and lanes C to E are UdECM25, UdECM50, and UdECM75 collagen samples, respectively. In Figure 2, the type I collagen standard and three UdECM collagen samples all showed a band at 245 kDa, which represents the β-chain, and two bands at 135 kDa (α1 and α2) with the formula [α1(I)]_2_[α2(I)]. This meant that the typical triple helix structure of collagen was similar to the type I collagen standard and the three UdECM samples. In summary, US-treated UdECM hydrogels were rich in type I collagen.

Collagen in UdECM samples was quantitatively analyzed with a hydroxyproline assay kit. Lyophilized-dried hydrogels of UdECM25, UdECM50, and UdECM75 were first hydrolyzed with a strong acid (10 N HCl), and we measured the total collagen content compared to those for a dECM_HCl_ hydrogel (25 mg/mL) (pepsin-mediated solubilization of dECM in a dilute HCl solution) and fresh porcine skin tissue (porcine) as control groups [17]. Results showed that the total content of collagen for the control group, dECM_HCl_ (25 mg/mL), UdECM25, UdECM50, and UdECM75 were 715.4 ± 7.34, 556.01 ± 5.9, 668.0 ± 0.8, 721.0 ± 0.7, and 757.0 ± 0.1 μg/mg dry weight, respectively. Collagen contents for the control group, UdECM50, and UdECM75 were in a similar range of 700–750 μg/mg dry weight, followed by the UdECM25 group with a collagen content of 650–700 μg/mg dry weight, while the dECM_HCl_ (25 mg/mL), extracted using pepsin solubilization in an HCl solution for two days, contained the least amount of collagen. This confirmed that US could produce cavitation between the solvent and sample, thereby increasing the dispersion and contact area of the collagen sample with enzymes in the extraction solvent. As a result, the extraction rate of collagen from the dECM sample is improved by the addition of ultrasonication.

#### 3.1.3. DNA Quantitative Analysis

DNA contents of those decellularized samples were measured compared to fresh porcine skin tissue, which was the control group, by following a previously reported procedure [17,27]. The results showed that following a 72 h decellularization process in 30% formic acid, the DNA contents of both dECM and UdECM were consistently reduced to 46.49 ± 0.79 and 42.95 ± 0.73 ng/mg dry weight, respectively. In comparison, fresh porcine skin soaked in PBS exhibited a much higher DNA content of 349.77 ± 8.33 ng/mg dry weight, resulting in a removal efficiency of nearly 80%. With a residual DNA content of less than the minimum allowance of 50 ng/mg dry weight, both dECM and UdECM had reached the minimum standard for decellularization. Furthermore, US was able to destroy residual cells to extract more DNA contents and reduce the DNA content of 46.49 ± 0.79 ng/mg dry weight for dECM to that of 42.95 ± 0.73 ng/mg dry weight for UdECM with statistical significance.

### 3.2. Rheological Studies

The Amplitude Sweep program in the intelligent advanced rheometer (MCR 102e Anton-Paar) was used to evaluate the rheological characteristics of these hydrogels, including UdECM25, UdECM50, and UdECM75. Changes in G′ and G″ in the range of 0.1–100 Pa were measured at 25 and 37 °C. If G′ > G″, the sample shows a gel-like or solid structure and can be termed a viscoelastic solid material. However, if G″ > G′, the sample displays fluid characteristics and can be termed a viscoelastic liquid. Results are presented as the diagram in which the storage modulus, G′, and loss modulus, G″, were respectively plotted on a logarithmic scale as the X-axis and Y-axis. The limit of the linear viscoelastic region (LVR) determined the stress range in which the test could be carried out without destroying the sample structure. A wide LVR means that the hydrogel has good strength with structural stability to resist high stress.

**Figure 3:**
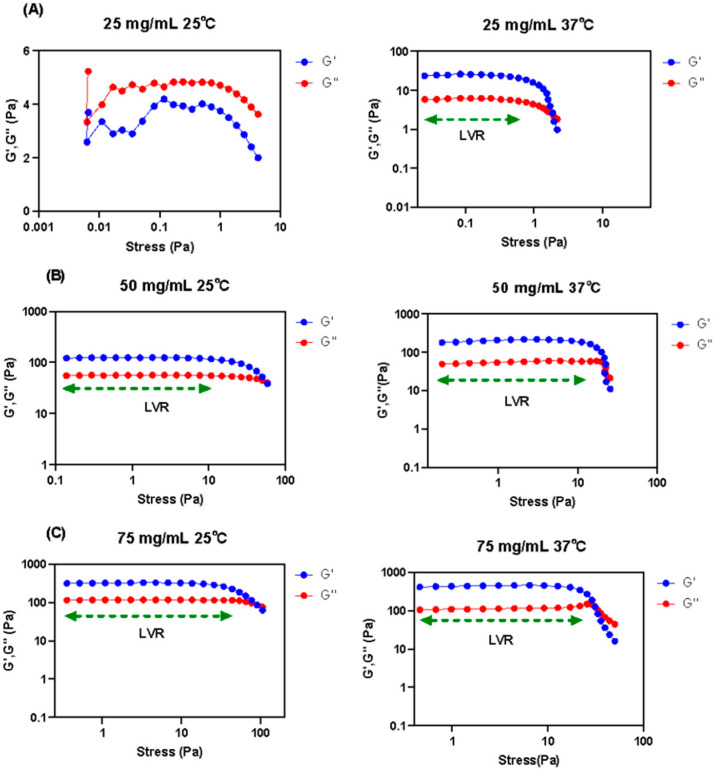
Viscoelasticity characterization of (**A**) ultrasound-assisted pepsin-solubilized decellularized extracellular matrix at 25 mg/mL (UdECM25), (**B**) UdECM50 (50 mg/mL), and (**C**) UdECM75 (75 mg/mL). Representative graphs of the UdECM hydrogel gelation kinetic profiles at two temperatures for three different concentrations of UdECM.

As shown by Figure 3A, when the temperature was 25 °C, G″ for UdECM25 was greater than G′, indicating a viscoelastic liquid state. However, at 37 °C, G′ surpassed G″, demonstrating a gel-like or solid structure. Nonetheless, the LVR was short, and the hydrogel structure was not strong enough to withstand high stresses. On the contrary, both UdECM50 (Figure 3B) and UdECM75 (Figure 3C) exhibited a gel-like or solid structure throughout the temperature range from 25 to 37 °C. Moreover, the hydrogel strength at 37 °C was higher than that at 25 °C, and the LVR was longer at 37 °C. These results revealed that with higher dECM concentrations than 25 mg/mL, the gel-like structure remained unchanged as the temperature increased from 25 to 37 °C as evidenced by G′ >> G″ being significantly greater than G″. Importantly, it was observed that a higher concentration of dECM contributed to a larger LVR and higher G, leading to a greater stiffness and structure stability of the hydrogel. However, other rheologic properties, including oscillatory deformation and steady shear deformation, were not evaluated in this study and need to be further investigated.

To the present, the reason for US being capable of gelling dECM is still unclear. Based on our research, it was assumed that collagen proteins were not the only self-assembling molecules in the UdECM mixture. Interactions among various components dissolved in UdECM might include other self-assembling molecules such as collagen, laminin, and proteoglycans. The above results were similar to a reference that ECM subjected to US could induce rapid hydrogelation below 25 °C [16].

#### Morphological Studies

**Figure 4:**
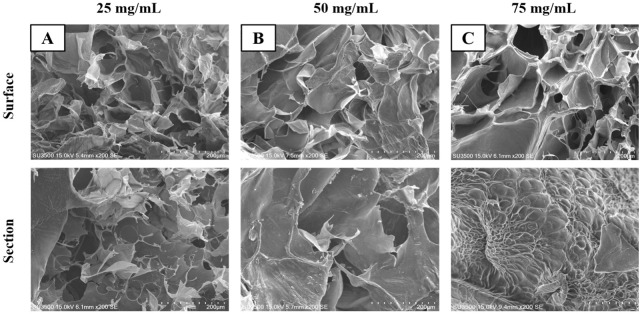
SEM images of the surface (top) and the middle section (bottom) of (**A**) ultrasound-assisted pepsin-solubilized decellularized extracellular matrix at 25 mg/mL (UdECM25), (**B**) UdECM50, and (**C**) UdECM75. Samples were imaged at 200× magnification (scale bars = 200 µm).

The UdECM25, UdECM50, and UdECM75 hydrogels were freeze-dried to form a spongy matrix. Furthermore, the density, porosity, and cross-section were observed by SEM. Figure 4 shows images of the surface (top) and cross-section (bottom) of (A) UdECM25, (B) UdECM50, and (C) UdECM75 spongy matrices. SEM demonstrated that all three spongy matrices were porous on the surface and had dense, rough, layered sheet structures on the cross-section. The denser cross-section with a less-porous surface increased the concentration of UdECM in the spongy matrix. The optimal porosity and density of the cross-section are critical to controlling the release of growth factors embedded in the UdECM hydrogels at desirable rates to promote utmost wound healing.

### 3.3. Contents of PDGF-AB and TGF-β1 in R-PRP

PRP, as a plasma product, was found in clinical applications to have large quantities of autologous growth factors, which are released after activation. These autologous growth factors play vital roles in cell growth and differentiation processes associated with wound healing [28]. Among them, TGF-β, VEGF, and PDGF-AB have the highest concentrations and can stimulate collagen synthesis, callus formation, and tissue regeneration. Thus, the PDGF-AB and TGF-β contents in PRP were used as indicators of the bioactivity of R-PRP to promote wound healing. After activation, the contents of PDGF-AB and TGF-β1 in R-PRP were 1103 ± 6.7 and 104 ± 5.0 ng/mL, while those for inactivated R-PRP were 30.2 and 23.3 ng/mL, respectively [29]. In conclusion, activation of PRP can boost the release of PDGF-AB and TGF-β1 growth factors from α-granules stored in platelet cells, and thus, the higher concentrations of both can enhance the repair ability and reduce wound-healing times.

### 3.4. In Vivo Testing on Diabetic Animals

**Figure 5:**
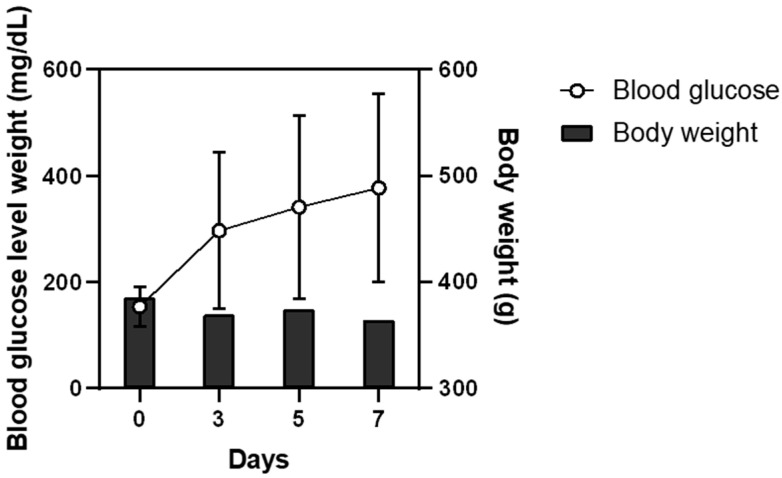
Blood glucose levels and body weights of a rat model of vitamin B3/streptozotocin (STZ)-induced diabetics. Values are presented as the mean ± SD (*n* = 13).

In diabetic animal experiments, 180 mg/kg of a 50% nicotinamide (Vitamin B3) solution was IP administered to rats in the various groups, and after 15 min, 65 mg/kg body weight (BW) of STZ was IP injected to produce an animal model close to type 1 diabetes. Figure 5 shows the blood glucose values and BWs on days zero, three, five, and seven after injections of Vitamin B3 and STZ. BWs slightly decreased on the third day during the induction period, and BWs were maintained at 300–400 g from the third to seventh days. After the drug was given, blood glucose values sharply rose on the third day and reached a peak on the seventh day. Because STZ can destroy β cells of animal islets, most of the pancreatic islet β cells in the rats were destroyed, which resulted in insufficient insulin secretion, and energy could not be converted after eating. Due to insufficient insulin, glycogen could not be inhibited. As the BW decreased, the blood glucose value continued to rise. Furthermore, these rats were found to have polyphagia, polydipsia, and frequent urination. Therefore, this experiment successfully established a rat model of diabetes.

In order to explore the application of UdECM as a scaffold for diabetic wound healing, an 8 mm tissue sampler was used to establish a wound on the back of diabetic rats, and a silicone sheet was sutured near the wound to avoid false wound-healing and wound measurement errors. Then six different hydrogel dressings of UdECM, UdECM/R-PRP, UdECM/SCNFs, UdECM/SCNFs/R-PRP, UdECM/TEMPO 050, and UdECM/TEMPO 050/R-PRP were randomly assigned to each wound of diabetic rats. The hydrogel dressings were changed on days 0, 3, 7, 10, 14, 16, and 20. Pictures were taken to record the wound healing, and we continuously monitored blood glucose levels and BWs.

**Figure 6:**
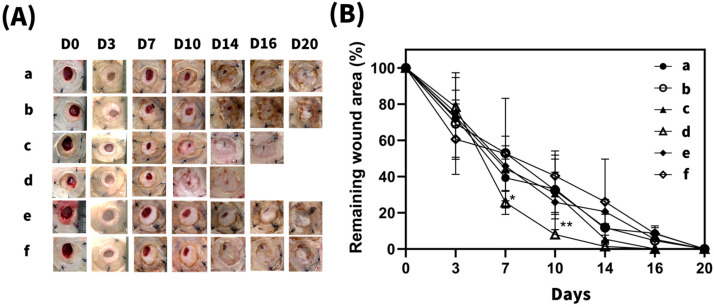
Wound-healing studies (*n* = 5 or 6) in diabetic rats. Patterns of the healing process vs. time (**A**) and the remaining wound area vs. time profiles (**B**) for six kinds of hydrogel dressings including (a) ultrasound-assisted pepsin-solubilized decellularized extracellular matrix (UdECM), (b) UdECM/rat platelet-rich plasma (R-PRP), (c) UdECM/sacchachitin nanofibers (SCNFs), (d) UdECM/SCNFs/R-PRP, (e) UdECM/TEMPO 050, and (f) UdECM/TEMPO 050/R-PRP. Values are presented as the mean ± SD (*n* = 5 or 6). * *p* ˂ 0.05, ** *p* ˂ 0.01 compared with UdECM/TEMPO 050/R-PRP group.

Figure 6 shows assessments of the wound area after 20 days of treatment by the wound appearance, and healing profiles were also calculated using the ImageJ image-processing analytical software. Figure 6A demonstrates that all wounds gradually healed without redness or ulceration. Among them, the UdECM/SCNFs/R-PRP group had completely healed by day 14, followed by the UdECM/SCNFs group (without R-PRP) that had healed by day 16, and the other groups (UdECM, UdECM/R-PRP, UdECM/TEMPO 050 and UdECM/TEMPO 050/R-PRP) which had healed by day 20. UdECM, UdECM/R-PRP, UdECM/TEMPO 050, and UdECM/TEMPO 050/R-PRP had effectively healed by day 20, which indicated that UdECM itself was relatively rich in original proteins and retained various cytokines after US, such as fibroblast growth factor and TGF, that could effectively help tissues regenerate. Among them, UdECM/SCNFs/R-PRP-treated wounds healed rapidly by the 14th day and revealed that its SC antibacterial efficacy was the same as that of UdECM, which was a mixed wound dressing with an external antimicrobial microenvironment and tissue growth factors.

As illustrated in Figure 6B, rapid healing began on day seven in all groups, and the degree of wound-healing for UdECM/SCNFs/R-PRP was 58% ± 0.6% compared to day 0, whereas that of UdECM/TEMPO 050/R-PRP was only 45% ± 0.1%, and there was a significant difference (*p* ˂ 0.05) between these two groups. Furthermore, the UdECM/SCNFs/R-PRP group showed wound-healing on day 10, which had reached nearly 90% (89% ± 0.7%), whereas the UdECM/TEMPO 050/R-PRP group was only at 59% ± 0.7% healing (*p* ˂ 0.005). This indicates that SCNFs were more effective than TEMPO 050. UdECM and R-PRP with rich growth factors can be combined to form homogeneous hydrogels to help enhance therapeutic effects.

**Figure 7:**
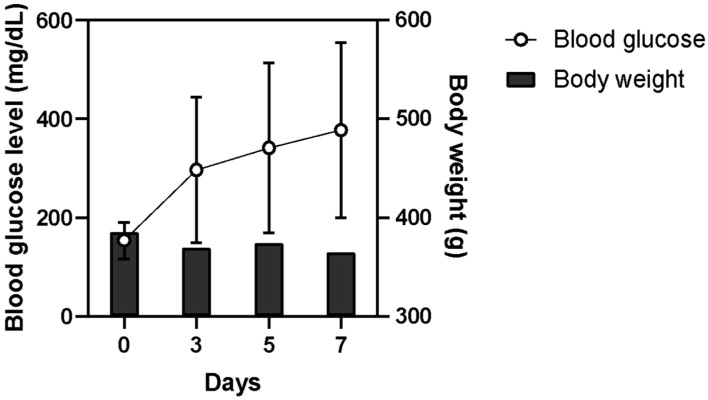
Determination of blood glucose levels and body weights in diabetic rats for 20 days. Values are presented as the mean ± standard deviation (*n* = 9–13).

Blood glucose levels and BWs were monitored during the treatment period to ensure that the rats continued to maintain diabetic disease patterns. As shown in Figure 7, during the follow-up period, blood glucose levels of diabetic rats continually remained higher than 350 mg/dL, and BWs remained at approximately 200 g, which confirmed that the rats were still hyperglycemic during the experiment.

The wound-healing test on euthanized diabetic rats was conducted on days 10 and 16. After removal of UdECM, UdECM/R-PRP, UdECM/SCNFs, UdECM/SCNFs/R-PRP, UdECM/TEMPO 050, and UdECM/TEMPO 050/R-PRP hydrogel scaffolds, skin tissues of the wound area were sectioned and stained with H&E, MT, and platelet endothelial cell adhesion molecule (CD31) for histopathological biopsies. H&E stain is a dual staining method for tissues that visualizes the distribution of nuclei (purple) and intracellular proteins (red). MT staining is used to observe the differential staining of collagen fibers and muscle fibers in connective tissue. Black indicates the nucleus, red the cytoplasm, keratin, or muscle fibers, and blue indicates collagen. CD31 is a surface marker for vascular endothelial cells, which are present in platelets, neutrophils, monocytes, and endothelial cells. Further, CD31 is commonly used to understand the distribution of neovascular growth.

**Figure 8:**
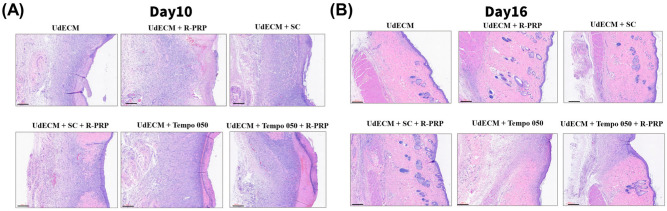
Hematoxylin and eosin (H&E)-stained images of ultrasound-assisted pepsin-solubilized decellularized extracellular matrix (UdECM), UdECM/rat platelet-rich plasma (R-PRP), UdECM/sacchachitin nanofibers (SCNFs), UdECM/SCNFs/R-PRP, UdECM/TEMPO 050 and UdECM/TEMPO 050/R-PRP hydrogel-treated wounds on days 10 (**A**) and 16 (**B**). (4×, scale bar = 300 μm).

Figure 8 illustrates H&E staining of skin tissues sampled on days 10 (Figure 8A) and 16 (Figure 8) after treatment with UdECM, UdECM/R-PRP, UdECM/SCNFs, UdECM/SCNFs/R-PRP, UdECM/TEMPO 050, and UdECM/TEMPO 050/R-PRP hydrogel scaffolds. Figure 8A shows that the newly grown tissue was in a red-dotted frame, and large numbers of cells were found to have accumulated around the wound in all treatment groups by day 10. This indicates that the wounds were gradually healing. Figure 8B shows the great improvement in wound healing with insignificant inflammation on day 16 in all treatment groups. As for dermal restoration, purple fibroblasts stained with H&E were uniformly dispersed in the dermis, which represented the transition of wound healing from the proliferation phase to the final tissue-remodeling stage. Compared to UdECM, UdECM/TEMPO 050, and UdECM/TEMPO 050/R-PRP hydrogel treatment groups, UdECM/R-PRP, UdECM/SCNFs, and UdECM/SCNFs/R-PRP treatment groups showed regeneration of new skin tissues in the epidermal layer. Obviously, epithelial tissue cells were neat, the blood vessel density of the wound had returned to normal, and the presence of hair follicles and sweat glands hinted at the significantly better healing efficacy of the latter three groups than the former three groups.

**Figure 9:**
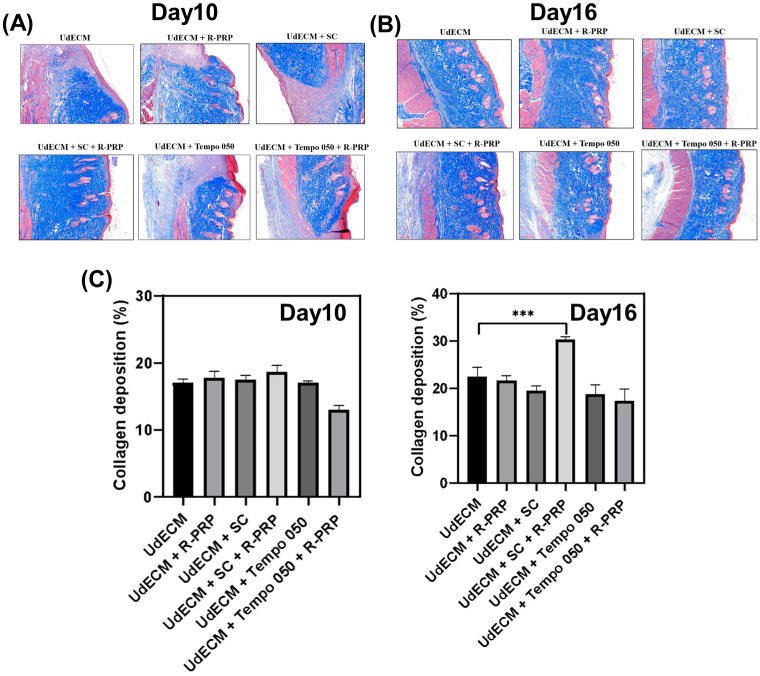
Masson’s trichrome (MT)-stained images of ultrasound-assisted pepsin-solubilized decellularized extracellular matrix (UdECM), UdECM/rat platelet-rich plasma (R-PRP), UdECM/sacchachitin nanofibers (SCNFs), UdECM/SCNFs/R-PRP, UdECM/TEMPO 050, and UdECM/TEMPO 050/R-PRP hydrogel-treated wounds on days 10 (**A**) and 16 (**B**). (4×, scale bar = 300 μm). Quantitative collagen deposition (**C**) based on MT staining of skin tissues sampled on days 10 and 16. *** *p* ˂ 0.005 compared to the UdECM group.

During the tissue-remodeling period, excess microvessels degenerated and became atrophied, the vascular density of the wound gradually returned to normal, collagen tissue was elongated and neatly arranged, and scars became flat and faded. To further assess wound healing, MT staining was used to confirm collagen recovery in regenerated tissues. As shown in Figure 9, MT staining for skin tissues sampled on days 10 (Figure 9A) and 16 (Figure 9B) are illustrated after treatment with UdECM, UdECM/R-PRP, UdECM/SCNFs, UdECM/SCNFs/R-PRP, UdECM/TEMPO 050, and UdECM/TEMPO 050/R-PRP hydrogel scaffolds. In Figure 9A, newly grown tissue appeared inside the black dotted frame, and there was a little collagen formed, which was irregularly arranged for the UdECM, UdECM/R-PRP, UdECM/SCNFs, UdECM/TEMPO 050, and UdECM/TEMPO 050/R-PRP treatment groups and which indicated that the wound had not completely healed. Exceptionally, the UdECM/SCNFs/R-PRP group was the only arm that showed collagen deposition and had gradually entered the tissue-repair phase.

Figure 9B illustrates MT three-color staining graphs for tissues sampled on day 16. From the area inside the black dotted frame, it was found that all treatment groups had large amounts of collagen deposition (blue-violet), but the UdECM, UdECM/SCNFs, UdECM/TEMPO 050, and UdECM/TEMPO 050/R-PRP treatment groups exhibited irregular arrangements of collagen in the dermal layer, which indicated that the tissues were still undergoing regeneration during the tissue-remodeling period. Obviously, the UdECM/SCNFs/R-PRP and UdECM/R-PRP hydrogel scaffolds demonstrated that the collagen in the dermal layer was neatly arranged and densely uniform, while UdECM/SCNFs/R-PRP showed the best wound-healing effect, as the tissue was completely repaired and remodeled by 16 days after treatment. Figure 9C illustrates the quantification results of collagen in tissue sections by image analytical software. The collagen content of the UdECM/SCNF/R-PRP treatment group had increasingly been deposited to 30% from days 10 to 16, whereas those for UdECM, UdECM/R-PRP and UdECM/SCNFs had moderately increased to 20–25%, and those for UdECM/TEMPO 050 and UdECM/TEMPO 050/R-PRP had slightly increased to less than 20%. This indicates that the use of collagen-rich UdECM could provide a good microenvironment for long-term wound repair. Furthermore, UdECM with the addition of either SCNFs (UdECM/SCNFs) and/or R-PRP (UdECM/R-PRP, UdECM/SCNFs/R-PRP) further synergistically enhanced diabetic wound-healing with greater collagen deposition.

**Figure 10:**
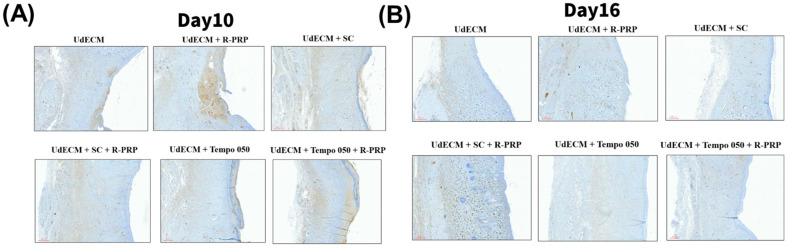
CD31 immunohistochemically stained (CD31) images of ultrasound-assisted pepsin-solubilized decellularized extracellular matrix (UdECM), UdECM/rat platelet-rich plasma (R-PRP), UdECM/sacchachitin nanofibers (SCNFs), UdECM/SCNFs/R-PRP, UdECM/TEMPO 050, and UdECM/TEMPO 050/R-PRP hydrogel-treated wounds on days 10 (**A**) and 16 (**B**). (4×, scale bar = 300 μm).

In immunohistochemical (IHC) staining, CD31 acts as the surface marker for vascular endothelial cells and is mainly used to indicate the presence of endothelial cell tissues and the distribution of neovascularization. As shown in Figure 10, CD31 was stained with brown dots or strips and was found to be evenly distributed in the dermal layer of skin tissues sampled on days 10 (Figure 10A) and 16 (Figure 10B). The brown dots or strips, as highlighted by red triangles in the figure, were new microvessels. On day 10, there was a large amount of CD31 expression in the UdECM/SCNFs/R-PRP group, which obviously indicated the formation of many new blood vessels. Figure 10B shows that expression in all groups significantly increased and was uniformly distributed from days 10 to 16. Furthermore, IHC staining of CD31 for the UdECM/SCNF/R-PRP and UdECM/R-PRP hydrogel treatment groups exhibited a more uniform and larger amount compared to the UdECM, UdECM/TEMPO 050, and UdECM/TEMPO 050/R-PRP groups. This indicated that more neovascularization had occurred in the dermis and also that excess microvessels were degenerating and atrophying to normalize the density of blood vessels in the wound. Thus, it was confirmed that the UdECM/SCNF/R-PRP and UdECM/R-PRP hydrogels greatly promoted the regeneration of blood vessels and provided sufficient nutrients to increase the ability of tissue repair and achieve wound healing.

In the comprehensive evaluation, wounds treated with different hydrogel scaffolds were scored based on scarring and the results obtained from H&E, MT, and CD31 staining on day 16. The detailed outcomes are presented in Table 2. The extent of wound healing was assessed using a grading scale ranging from 1 to 5, depending on the level of tissue remodeling observed (5: closest to normal skin appearance after treatment; 1: least resemblance to normal skin). The total score for normal skin was 20, indicating the highest level of recovery and resemblance to normal skin. Results in Table 2 show that the UdECM/SCNFs/R-PRP treatment group had the most significant efficacy for promoting the diabetic wound-healing process with a total score of 18, which meant the recovery was closest to normal tissues, followed by the UdECM/R-PRP and UdECM/SCNFs groups with similar total scores of 13–14. Additionally, UdECM hydrogel scaffolds with a total score of nine seemed to have only recovered to a 50% resemblance of normal skin. Treatment with the UdECM/TEMPO 050 and UdECM/TEMPO 050/R-PRP hydrogel scaffolds that combined UdECM with TEMPO 050, ranked the last with total scores of 5–7, which was even poorer than the UdECM hydrogel.

Several studies have demonstrated that ECM hydrogels effectively facilitate cell infiltration, with a particular emphasis on macrophages and progenitor cells. Furthermore, these hydrogels also promote neovascularization and exhibit positive functional remodeling, further highlighting their potential in tissue repair and regenerative applications [3]. This is the reason that UdECM hydrogels can moderately promote diabetic wound healing. Further, composite hydrogels composed of UdECM with SCNFs (UdECM/SCNFs) or R-PRP (UdECM/R-PRP) accelerated wound-healing faster than only UdECM hydrogels (UdECM). The observed potential mechanism underlying UdECM/SCNF hydrogels’ efficacy may be attributed to two main factors: rapid re-epithelialization and normal ECM deposition. These hydrogels create a moist microenvironment that fosters enhanced cellular movements, promoting an accelerated healing process. Furthermore, the inclusion of SCNFs in the hydrogel formulation may contribute to a chemotactic effect on inflammatory cells, facilitating angiogenesis and promoting the formation of granulation tissue. Ultimately, this synergistic effect leads to the formation of new tissue, thereby contributing to the overall improvement in diabetic wound healing [30,31]. Obviously, combination therapy of UdECM hydrogels with R-PRP (UdECM/R-PRP) accelerated diabetic wound-healing because of greater amounts of growth factors, such as PDGF, VEGF, TGF-β, and EGF, which can inhibit inflammatory symptoms and promote the formation of microvessels and epithelialization of chronic wounds.

Obviously, UdECM/SCNFs/R-PRP showed the greatest potency for diabetic wound healing. Notably, the diabetic wounds treated with UdECM/SCNF/R-PRP hydrogels achieved complete healing within 14 days, which closely resembles the healing timeline observed in our prior study where diabetic wounds were treated with aECMHCl,25/SC hydrogels and also fully healed within 14 days [17]. These findings underscore the remarkable therapeutic potential of UdECM/SCNF/R-PRP hydrogels and their efficacy in promoting efficient diabetic wound closure. However, Hung et al. reported that skin wounds covered with an SC membrane needed 21 days to heal [32]. This discrepancy might be attributed to synergistic promotion by the composite hydrogel and the incorporation of enriched amounts of several growth factors. Furthermore, compared to SC membranes, the design of UdECM/SCNF/R-PRP hydrogels aims to create a moist environment that fosters optimal wound-healing conditions. With a high moisture content exceeding 90% water, UdECM/SCNFs/R-PRP hydrogels effectively contribute to impeding bacteria from accessing the wound site, acting as a protective barrier against potential infections. This unique characteristic enhances the hydrogel’s ability to support the healing process and leads to improved wound closure outcomes [33]. It was concluded that UdECM hydrogels could be prepared by US digestion, which has the advantage of rapid and effective preparation for type I collagen-rich hydrogels from formic acid-decellularized ECM (dECM). In in vivo tests, UdECM/SC composite hydrogels loaded with PRP could heal diabetic wounds in 14 days. In histopathological sections, UdECM/SCNFs/R-PRP effectively promoted diabetic wound healing and angiogenesis. Overall, UdECM/SCNF/R-PRP hydrogels controlled the release rate of platelet-derived growth factors and retained the antibacterial ability of SC, thereby shortening wound-healing times.

## 4. Conclusions

In this study, ultrasound-assisted digestion of decellularized extracellular matrix (UdECM) was successfully developed. Composite hydrogels composed of UdECM hydrogels and SCNF hydrogels or UdECM hydrogels incorporated with R-PRP improved diabetic wound healing faster than UdECM hydrogels. UdECM/SCNFs/R-PRP demonstrated the greatest enhancement of diabetic wound healing. In conclusion, UdECM hydrogels developed in this study combined with SCNF hydrogels and/or incorporated with R-PRP became a convenient and effective hydrophilic dressing, which ultimately has a wide range of applications for wound-healing and regenerative medicine.

## Figures and Tables

**Table 1:** Formulations of wound dressings.

	A	B	C	D	E	F
Wound Dressing	UdECM	UdECM/PRP	UdECM/SCNFs	UdECM/SCNFs/PRP	UdECM/TEMPO 050	UdECM/TEMPO 050/PRP
UdECM (mg)	50	50	50	50	50	50
SCNFs (mg)			50	50		
TEMPO 050 (mg)					50	50
R-PRP (pg)		800		800		800

Abbreviations: UdECM, ultrasound-assisted pepsin-solubilized decellularized extracellular matrix; SCNFs, sacchachitin nanofibers; TEMPO 050, TEMPO-oxidized sacchachitin nanofibers; R-PRP, rat platelet-rich plasma.

**Table 2:** Overall assessment of the in vivo wound-healing study on day 16. The degree of wound healing was graded from 1 to 5.

Wound Dressing	Scarring	H&E	MT	CD31	Sum
UdECM	1	2	4	2	9
UdECM/R-PRP	3	3	4	4	14
UdECM/SCNFs	3	4	3	3	13
UdECM/SCNFs/R-PRP	4	5	5	4	18
UdECM/TEMPO 050	1	1	2	1	5
UdECM/TEMPO 050/R-PRP	1	2	2	2	7

Abbreviations: UdECM, ultrasound-assisted pepsin-solubilized decellularized extracellular matrix; R-PRP, rat platelet-rich plasma; SCNFs, sacchachitin nanofibers; TEMPO 050, TEMPO-oxidized sacchachitin nanofibers; H&E, hematoxylin and eosin; MT, Masson trichrome; CD31, cluster of differentiation 31.

## Data Availability

Source data are available from the corresponding author upon reasonable request.
